# Veneer Inlay

**Published:** 1908-01-15

**Authors:** W. Clyde Davis

**Affiliations:** Lincoln, Neb.


### VENEER INLAY.

BY W. CLYDE DAVIS, M.D., D.D.S., LINCOLN, NEB.

I wish to call the attention of the dental profession to an inlay which might be called a veneer inlay, some of the features of which, especially as to methods of construction, I believe to be original.

Whenever something new is taken up by our profession we are likely to attempt to apply it in every case presented and not always with the best of results. This is true of the craze for inlays of all kinds, yet we are setling down to their application in their legitimate field.

It is probably true that there is not yet before us a better filling material than pure gold, for undoubtedly a greater percent of permanent fillings have been made of this material than any other, due largely to its ductility, whereby we may burnish its margins to a perfect adaptation, whether it be a built-in filling of gold alone or a combination with cement, or the cemented inlay.

The legitimate field for gold inlays will eventually be in cases where a large amount of tooth substance has been lost; and, if they finally become of universal use they must be demonstrated to be more permanent than the built-in filling, they must take less time of both patient and operator, and if they require less gold it will add materially to their popularity.

These advantages can never be attained in the case of small fillings, especially those where convenient access is difficult to obtain, not even with the cast inlay, for many are the operators who can and do insert a small gold filling, since the introduction of quick-working golds, in very little more time than it would require to make and invest the wax mold for a cast inlay, which is without doubt the quickest method yet before us for inlays. Should we insist on the virtues of a cemented joint these operators would reply: "We would first give the cavity the usual mechanical retention and build the filling into a film of soft cement, and we have your inlay beaten in every point as to speed, retention and certainly it is as lasting, for we have retention in all directions."

But when it comes to large restorations it is different, as the fused inlay can be more quickly made than a filling with a plugger point, hence less tiresome for both patient and operator; and, by the method given below, with about one-third to one-fourth the gold generally used either in the inlay or the built-in filling. In cases where the teeth are commonly crowned the filling should weigh scarcely more than a shell gold crown.

This filling may be made either by the sweating process upon a matrix or the casting process, and for a cavitv that has all of the retentive form and unevenness of outline ever desired or met with in the usual procedures in any built-in metal filling.

Take any large cavity, wherever located, either simple or complex, prepare the cavity in the usual way as for the reception of an amalgam restoration, except the cavo surface may be more extensive if desired and the cavo surface angle more obtuse because of the superior edge strength of the inlay. The cavity should be retentive in all directions and may possess acute line and point angles. Next warm a sufficient quantity of "Metalline" (a new substance to be had at most dental depots) and pack the cavity with same until you have, as a rule, filled all that part of the cavity formerly occupied by dentin. Circumstances and the taste of the operator would modify this amount in some cases, but as a rule that should be about the amount. This will leave a remaining cavity, for which it is an easy matter to make a matrix, if that method is to be used. Before burnishing matrix or taking impression the Metalline should be flooded with a jet of cold water from the syringe. When the matrix has been superficially burnished it should be removed, coated with whiting on cavity side, and the external surface covered with a thin film of pure gold, then returned to cavitv and finally burnished. Patient may be dismissed with the Metalline in the tooth and return for final setting at a subsequent time. Again coat cavity side of matrix and proceed to sweat up to proper contour as to cusps and contact point. The result will be a veneer of gold not unlike the shape of the lost enamel.

Next solder a suitable square platinoid bar, either straight curved or looped at two advantageous points so that it will pass through the body of the cavity.

When the patient returns, warm the Metalline in the tooth with a blast of warm air, entirely remove, fill cavity to overflowing with cement and crowd inlay to place, by means of one or two sharp blows with a mallet on a wooden stick. Should your matrix have been a slight misfit, as all that I have ever seen are, there will be sufficient give to the margins to settle the inlay to a close adaptation.

By the casting process there is no change up to the point of using the matrix. When the Metalline has been placed in position, restore the remaining portion with the special wax furnished with the casting outfit. When fully carved remove and put into position your platinoid bar, pin or staple, whichever suits the case, securing same with wax at its ends. Bar should be slightly warmed so that it will settle a slight distance into the wax. Invest and cast in the usual manner and filling will come out with bar in proper position. Those who desire may leave out bar and solder into position, as in the case with the matrix inlay, or in place of the bar, form a small roll of the casting wax to the desired shape and attach to the wax inlay at its two ends the same as with the bar. This will cast the same as the inlay. This plan will not alloy the pure gold should you desire to recast, and is the best method when giving table clinics, and the inlay is frequently refused.

When completed, as described before, the result is an inlay of sufficient strength to sustain a bridge. It rests in a cavity of the usual retention and the cement around the bar is more than ample. The quantity of gold used is materially lessened, a point not to be ignored by the frugal.

Lastly, should the pulp canals subsequently need attention you have not a large mass of gold to penetrate in order to reach them, a point one will appreciate if perchance, they have ever gone through a solid inlay over an inflamed pericemental membrane.—December *Dental Digest.*

